# Association of parental social support and dental caries in hispanic children

**DOI:** 10.3389/froh.2023.1261111

**Published:** 2023-11-15

**Authors:** Casey Lally, Nihmath Nasiha Maliq, Madysen Schreiber, Anne Wilson, Tamanna Tiwari

**Affiliations:** ^1^ Children’s Hospital, University of Colorado, Aurora, CO, United States; ^2^ School of Dental Medicine, University of Colorado, Aurora, CO, United States; ^3^ School of Public Helath, Oregon Health and Sciences University, Portland, OR, United States

**Keywords:** parental, social, support, dental, caries, hispanic, children

## Abstract

This study describes social support of Hispanic parents and the correlation with dental caries in their children. A cross-sectional study design was utilized to assess the 157 parent-child triads recruited from the Children's Hospital Colorado Dental Clinic. The Basic Research Factors Questionnaire (BRFQ) survey was utilized to assess parents' oral health knowledge, attitudes, behavior, and other psychosocial measures with social support as the main predictor variable. Bivariate associations between the independent variables and dmfs were conducted. Independent variables with a bivariate association of *p* ≤ 0.2 for the outcome variable were included in the multivariable linear regression model. Dental caries in children was significantly associated with less overall parental social support (*β* = −10.10, *p* = 0.03). Overall social support was divided into four sub-categories: errand help, money help, childcare help, and transportation help. Dental caries decreased by 7.70 units for every 1-unit increase in transportation help (*β* = −7.70, *p* = 0.03). A significant association was observed between parental knowledge on dental utilization and dmfs (*β* = −2.70, *p* = 0.04). In the multivariable linear regression model, caries was significantly associated with social support (*β* = −11.18, *p* = 0.02) and knowledge on dental utilization (*β* = −3.84, *p* = 0.01). The study concludes that a higher level of social support and knowledge on dental utilization for Hispanic parents is correlated with lower rates of dental caries in their children.

## Introduction

According to the Oral Health in America report, 1 in 4 preschool-aged children have experienced dental caries in their primary teeth, and 1 in 6 children aged 6–11 years have experienced dental caries in their permanent dentition (1). Significant disparities exist in the prevalence of dental caries among children of lower socioeconomic status and different race/ethnic groups. Hispanic preschool children are reported to have higher rates of dental caries on average than non-Hispanic white children (1). Hispanic or Hispanic Americans include people of Cuban, Mexican, Puerto Rican, South or Central American, or other Spanish culture/origin, regardless of race (2). As of 2020, Hispanics make up 19% or 62.1 million Hispanics in the United States (3), and children within this population experience disproportionate rates of dental caries compared to non-Hispanic white children in the United States (1). According to the CDC's Oral Health Surveillance Report, 33% of Mexican-American children aged 2 to 5 years old experience dental caries in their primary teeth, compared to 18% of non-Hispanic white children (4). In older children, 70% of Mexican American children ages 12 to 19 years old experience caries in their permanent teeth compared to 54% of non-Hispanic white children (4).

Oral disease can contribute to negative psychosocial sequelae involving children, parents, family, and the community. These impacts, which include factors like social environment and individual mental health, can reduce the quality of life and lead to outcomes such as orofacial pain and infection, reduced effectiveness at school or work, and increased financial burden (5, 6). Psychological stress can be related to oral health through direct and indirect mechanisms (7). Directly, neuro-endocrine immune stress affects host defenses (8). Indirectly, psychological stress can increase the chance of developing health-compromising behaviors (9). A recent systematic review reported that “protective psychosocial factors are associated with greater frequency of toothbrushing, lower consumption of sucrose, higher frequency of dental visits, and dental checkups” (7). This review also found that parental self-efficacy and social support are associated with dental caries in their children (7).

Social support, which is defined as “an exchange of resources between at least two individuals perceived by the provider or recipient to be intended to enhance the well-being of the recipient”, has been studied for its relationship with overall health (10). The underlying mechanism of action that supports the relationship of social support to health has conceptualized that social support helps individuals to cope with the stresses of life (11). Individuals with strong social support are thought to be more able to cope with stressful life events than those with weaker social support (12). Social support can impact health by affecting knowledge, thoughts, attitudes, and behaviors so as to promote health outcomes (13). Social support, whether it be positive or negative relationships, has been shown to correspond to the level of health and health outcomes. Low levels of social support have been shown to negatively affect patients with coronary artery disease (14). Studies have also shown that social support could play a role in the genetic pathogenesis of clinical depression and that higher levels of social support could moderate the risk of depression (15). These various studies have shown that when assessing the overall health of an individual, social support cannot be ignored.

Social support plays an important role in oral health outcomes. Several studies have shown a relationship between social support with dental caries and dental care utilization. A study by Nahourrai et al. focused on social support among Latina immigrants (16). In this study, social support was divided into four subtypes (information, influence, material aid, and emotional aid), and associations between dental use among children of Latina immigrants were tested (16). A total of 65.2% women enrolled in the study received at least one of the types of social support (16). While information alone was not found to be associated with dental care use, receiving any of the other types of social support was associated (16). Hispanic mothers with social support from family or friends had a 3 times greater chance of using dental services for their children, thereby concluding that social support was significantly associated with the child's dental care use (16). According to a cross-sectional study by Fontanini et al., low numbers of social networks and low levels of support from family in adolescents were associated with a DMFT ≥ 1 and current caries (17). Similarly, a retrospective cohort study by Nelson et al. explored maternal enabling factors, stress, coping, and social support in relation to adolescent dental visits and DMFT (18). Social support was measured by marital status and the multidimensional scale of perceived social support, which assessed perceived social support from family, friends, and significant others (18). A higher score on this scale indicated that the mother had more resources to manage stress (18). Notably, this study revealed that higher maternal social support when the child was age 3 was associated with lower DMFT in adolescence (18). Mothers reporting greater social support were 28% less likely to have a child with a higher mean DMFT (18). Another study by Burgette et al. focused on evaluating Northern Appalachian mothers' perceived level of social support and the probability of high dental caries levels in their children (19). In this study, social support was divided into four domains (appraisal, tangible, self-esteem, and belonging), and found that appraisal support, or the mothers' perception of having someone to talk to about problems, was associated in a lower probability of dental caries in their children (19). These studies all contribute to the understanding that social support is an influential psychosocial factor when it comes to the development of dental caries in children. This study aims to investigate the impact parental social support and knowledge of dental utilization have on dental caries in Hispanic children. We hypothesize that there will be an inverse relationship between parental social support among Latino parents and the prevalence of dental caries in their children.

## Methods

### Study design and sample size

This study (17-0041) was approved by the Colorado Multiple Institutional Review Board (COMIRB). The primary study was conducted with 200 parent-child triads whose children were patients at the Dental Center at the Children's Hospital Colorado and Salud Family Health Centers. The sampling technique used was a type of non-probability sampling, more specifically, convenience or purposive sampling. Study enrollment began in July 2017 and concluded in March 2019. The triads consisted of a Hispanic primary parent, at least 18 years of age, with two children, one between 0 and 6 years and the second between 0 and 10 years of age. The primary results of the study were published which provide more details on the study design (20). Parents were included in the study regardless of primary language spoken, English or Spanish, and those with missing data for social support items were excluded, resulting in an analyzed sample of 157 parent-child triads. A cross-sectional study design was utilized where the prevalence of dental caries in primary dentition was assessed in the study population.

### Data collection

The parent was approached by a member of the research team in the waiting area of the dental clinic to explain the study procedure. Spanish-speaking parents were provided information about the study by certified translators. All parents were given a Basic Research Factors Questionnaire (BRFQ) (21) survey in Spanish or English, depending on their primary language. Those who agreed to participate in the study signed the consent form prior to completing the questionnaire. The survey was completed in English/Spanish based on the parents' language of preference using an iPad and REDCap (Research Electronic Data Capture) electronic data capture tool hosted at the University of Colorado Anschutz Campus. REDCap is a secure, web-based software supporting clinical research data capture.

### Basic research factors questionnaire (BRFQ) survey

The BRFQ was developed through a collaborative effort between three Centers for Research to Reduce Oral Health Disparities Centers: University of Colorado Anschutz Medical Campus, Boston University, and University of California San Francisco funded by the National Institute of Dental and Craniofacial Research. It is a computerized 190-item questionnaire that assesses parents' oral health knowledge, attitudes, behavior, and other psychosocial measures in addition to collecting parent and child socio-demographic characteristics (21). The BRFQ's validity was rigorously addressed through a comprehensive expert-led development process, ensuring content, construct, and cultural appropriateness (21). BRFQ is available in English, Spanish and few other languages and is used in several populations, including low-income, underserved families, particularly Latinos and non-native English speakers in San Francisco; low-income, diverse, underserved families living in urban, public housing developments in Boston; and American Indian (AI) families living on Reservations. BRFQ has been completed by over 5,000 participants. Survey-related fatigue has not been reported by the study participants, and studies revealed no patterns suggesting effects on data of fatigue from response burden (22).

### Variables

The outcome variable for the investigation is a continuous variable known as “decayed, missing, and filled tooth surfaces measure” (dmfs). Dental caries was identified by cavitated lesions, characterized either from evident fractures in the enamel or noticeable, irreversible enamel loss (23, 24). The clinical data was collected by two providers. A study-trained and calibrated licensed dentist and a dental hygienist conducted the visual screenings of the enrolled children to score dmfs. The dentist calibrated the dental hygienist each year, and interrater reliability was logged. Examinations were conducted using a mouth mirror and an overhead light attached to the dental chair using the method described by Pitts (25). The findings were input into an electronic dental research record system called Caries Research Instrument (CARIN).

#### Social support

Social support was the main independent predictor variable indicating the degree to which parents believe they have others available to help when needed. Social support was addressed by four item questions reflecting whether a parent reported support to: (a) run their errands, (b) help amid a financial crisis, (c) watch their child, and d) help with transportation when needed. The overall social support score is computed as the average score of all the items.

### Secondary independent predictor variables include the following

#### Oral health behavior

Twelve items were used to obtain an overall behavior score that was represented by the percentage of oral health behavior items answered with an “adherent” response. Adherent is defined as following recommended oral health behavior. The 12 questions assessed oral health behaviors with regard to oral hygiene and diet.

#### Oral health knowledge

Fourteen items were used to assess parent knowledge related to oral hygiene routines and feeding practices. The responses were coded as correct or incorrect, and an overall knowledge score was calculated as a percentage of questions answered correctly.

#### Self-efficacy

Ten items assessed parents' confidence in the ability to successfully engage in recommended oral health behaviors for the child. The average of the responses was calculated for each participant, with larger numbers representing a greater degree of self-efficacy.

#### Knowledge on dental utilization

Five items were used to measure parents’ knowledge on utilization of oral health services. The average of the scores was calculated as the overall score. In addition, each item was assessed independently with dmfs.

#### Parent stress index

Nine items measured perceived stress related to the caregiving role, and the overall score was computed as the average score of all the items.

#### Chronic stress

Eight items measured the parents' stress related to situations in everyday life. The overall chronic stress score was computed as the average score of all the items.

#### Acculturation

The twelve-item Acculturation Rating Scale for Mexican Americans (ARSMA-II) was used to measure acculturation. The responses were averaged across items for the overall acculturation score.

#### Health belief model

Sixteen items measured four key concepts of the health belief model. Key constructs included perceived susceptibility, perceived seriousness, perceived benefits, and perceived barriers. Responses ranged from 1 (strongly disagree) to 5 (strongly agree) and the average of the items associated with each construct was computed. Items under perceived barriers are reverse coded.

#### Parent and child socio-demographics

Demographic data was collected on parents’ age, education, employment status, household size, number of minors in the household, the number of years the family has lived at the current residence, and the language of preference.

### Statistical analysis

The outcome variable dental caries (measured by dmfs) is recorded using one reference child from each family. Descriptive statistics for the continuous variables are summarized with means and standard deviations and the categorical variables are summarized with counts and percentages to ascertain the characteristics of the study sample. Bivariate associations between each of the independent variables and “dmfs” were conducted using simple linear regression analysis. The associations between the four individual questions for social support and dental caries (measured by dmfs) were also modeled using simple linear regression analysis. All of the independent variables with a bivariate association of *p* ≤ 0.2 with the outcome variable were included in the multivariable linear regression model to assess the simultaneous, independent association between each variable and dental caries. Estimates, 95% confidence intervals, and *p*-values are reported. All data cleaning and analyses were conducted using SAS version 9.4.

## Results

The sample size of this study is 157 parent-child triads. Table 1 provides descriptive statistics of patients enrolled in the study with categorical variables summarized as frequencies and percentages and continuous variables summarized as means and standard deviations. The mean age of children enrolled in the study was 6.36 years (SD = 2.54 years), and the mean age of parents enrolled in the study was 29.86 years (SD = 9.71 years). Of the children enrolled in the study, 80 children were reported as male and 77 children as female. The average dmfs score for children enrolled in the study was 12.85 (SD = 17.48). Among the parents in this study, 63.39% are unemployed, and 43.31% have less than a high school education. Parents reported an average household size of 5.38 (SD = 1.76) with an average of 3.08 (SD = 1.30) minors in the household. The mean value for knowledge on dental utilization was 3.51 (SD = 1.07), and the mean value for the parental characteristic of social support was 1.18 (SD = 0.32).

**Table 1:** Descriptive statistics; categorical variables summarized as frequencies (percentage); continuous variables summarized as mean (standard deviation).

Variables	Value (*n* = 157)
Child characteristics	
Dental caries (measured by dmfs)^a^	12.85 (17.48)
Child Age	6.36 (2.54)
Child Gender	
Male	80 (50.96%)
Female	77 (49.04%)
Parent characteristics	
Parent Age	29.86 (9.71)
Parent Education	
Less than high school	68 (43.31%)
At least high school	89 (56.69%)
Parent Employment	
Employed	57 (36.31%)
Unemployed	100 (63.39%)
Household Size	5.38 (1.76)
Minors in Household	3.08 (1.30)
Years in Household	
Less than 5 years	104 (66.24%)
At least 5 years	53 (33.76%)
Insurance	
Yes	125 (80.13%)
No	20 (12.82%)
Knowledge on Dental Utilization	3.51 (1.07)
Social Support	1.18 (0.32)

^a^ dmfs, decayed missing filled surfaces.

Table 2 provides the bivariate associations between predictors (parental social support, knowledge on dental care utilization, and parent stress index) and the prevalence of dental caries in children. These estimates and *p*-values are determined using bivariate linear regression analysis, and statistical significance is defined as *p* < 0.05.

**Table 2:** Association between parental social support, knowledge on dental care utilization and parent stress index and dental caries in children.

Variables	Estimate*	*P*-value*
Overall social support**	−10.10	0.03
Errand help (1 = yes)	−8.07	0.07
Money help (1 = yes)	−6.93	0.05
Childcare help (1 = yes)	−6.78	0.11
Transportation help (1 = yes)	−7.70	0.03
Knowledge on dental utilization	−2.70	0.04
Parent stress index	−2.53	0.01

* Estimates and *p*-values reported using bivariate linear regression analysis.

** Social support measured using four instruments, errand help, money help, childcare help, and transportation help.

A significant inverse association was observed between overall parental social support and the prevalence of dental caries in children (*β* = −10.10, *p* = 0.03). Specifically, for every 1-unit increase in overall social support, there was a decrease in dental caries, suggesting that higher levels of parental social support were associated with lower rates of dental caries among children. Thus, the findings corroborate the initial hypothesis, confirming the anticipated inverse relationship. This relationship also held true when parental social support in terms of transportation assistance was considered. For every 1-unit increase in transportation help, there was a significant decrease in dental caries in children (*β* = −7.70, *p* = 0.03). Additionally, there was a significant inverse relationship between parental knowledge on dental utilization and the prevalence of dental caries (*β* = −2.70, *p* = 0.04). This implies that parents who are more knowledgeable about dental health tend to have children with a lower prevalence of dental caries. Lastly, there was a significant negative correlation between parental stress and dental caries rates in children. For every unit increase in the parental stress index, there is a consequential decrease in the prevalence of dental caries in children, which underscores the important role parental stress plays in children's oral health outcomes.

In Table 3, a multivariable linear regression model analysis presents the association between parental social support and dental caries in children, adjusting for knowledge on dental care utilization and the parent stress index. In this adjusted model, the prevalence of dental caries in children was significantly associated with parental social support and knowledge on dental utilization. Every 1-unit increase in overall parental social support correlated with a significant decrease in the rate of dental caries in children (*β* = −11.18, *p* = 0.02). Likewise, a 1-unit increase in parental knowledge on dental utilization was associated with a reduction in the prevalence of dental caries in children (*β* = −3.84, *p* = 0.01). These results reaffirm that higher levels of parental social support and knowledge about dental care utilization can have a positive impact on reducing the prevalence of dental caries in children.

**Table 3:** Association between parental social support and dental caries in children adjusting for knowledge on dental care utilization and parent stress index.

Variables	Estimate*	*P*-value*
Overall social support	−11.18185	0.02
Knowledge on dental utilization	−3.84791	0.01

* estimates and *p*-values reported using a multivariate linear regression analysis.

Overall model *p*-value: 0.004.

## Discussion

This study contributes to our understanding of the association between social support and dental caries rates in Hispanic children, highlighting the role of parental social support in promoting oral health. The findings revealed a significant association between higher levels of social support among Hispanic parents and a reduction in dental caries in their children, supporting our hypothesis. Moreover, the study demonstrated that a higher level of social support and knowledge on dental utilization among Hispanic parents correlated with better oral health outcomes for their children.

Specific sub-categories of parental social support were examined, including assistance with errands, financial support, childcare, and transportation. Among these, transportation support was found to be particularly significant, with children experiencing lower rates of dental caries when their parents reported having access to transportation assistance. This finding highlights the importance of overcoming transportation barriers to ensure better access to dental care for Hispanic children. These results align with previous research, such as the study by Nahouraii et al., which explored dental utilization among children of Hispanic immigrants in North Carolina (16). Their findings demonstrated that parental social support significantly influenced dental care use, with higher odds of dental visits observed for children whose mothers had some form of social support (16). Similar results were reported by Iida et al., who found that mothers with low social capital were more likely to report unmet dental care needs for their children (26). These studies collectively emphasize the crucial role of social support in promoting dental care utilization and oral health outcomes in children.

Additionally, parental stress has been identified as another significant determinant of children's oral health, particularly influencing the prevalence of early childhood caries (ECC) in multicultural communities, such as Latinos (27). Findings of this study showed a notable negative correlation between parental stress and the prevalence of dental caries in children. To explain this unexpected finding, one can draw upon psychological phenomena, such as overcompensation (28). This concept implies the conversion of a perceived weakness into a strength, suggesting that increased parental stress might drive parents to be particularly attentive to their children's oral health as a means to counterbalance perceived inadequacies elsewhere (28). Furthermore, the potential influence of external factors cannot be ignored. There may exist interventions or programs that specifically target highly stressed families, offering enhanced dental care or support, which could bias the results. Similarly, a reporting bias might be at play. Parents having heightened stress might be less forthcoming about dental caries or more proactive in seeking early dental care for their children, leading to a diminished observed prevalence of caries in the research. Maternal self-efficacy, which may be affected by stress levels, plays a critical role in managing a child's oral health. The study by Wilson et al., found that a higher level of maternal self-efficacy correlated with lower instances of ECC in Latino children (27). This relationship underscores the importance of empowering parents with the knowledge and resources necessary to navigate their children's oral health effectively.

In addition to parental self-efficacy, social support structures have a profound influence on a child's oral health-related quality of life over time (29). By fostering supportive environments, stress levels can be reduced and self-efficacy improved, resulting in better oral health outcomes for children (29). Furthermore, the results of our research underscore the significant impact of parental knowledge on children's oral health. This is particularly salient in multicultural contexts where parental beliefs and knowledge might diverge from mainstream oral health practices (30).

Moreover, responses to the current study's survey regarding parental knowledge of dental utilization provide insights into potential barriers Hispanic parents might encounter. Findings suggest that parents of Hispanic children with dental caries may exhibit lower functional health literacy, potentially impeding their navigation of the United States healthcare system. Language proficiency and communication challenges with healthcare providers also contribute to limited access to oral health services. Similarly, a study conducted by Barker et al. delved into the contextual factors surrounding parental knowledge and familial beliefs concerning oral health and dental care for children (31). Using open-ended questions, the study aimed to gain insights into the parents' perspectives on access to and utilization of oral health care services in the region (31). The findings revealed that Hispanic parents possess a strong desire for their children to maintain good health and exhibit a keen willingness to enhance their oral health status (31). However, the study illuminated a knowledge gap and resource constraints that impede their ability to effectively address their children's oral health needs (31). Therefore, interventions aimed at bolstering parental knowledge, enhancing social support, and reducing parental stress could be effective strategies for improving children's oral health, particularly in vulnerable populations. These multifaceted approaches are likely to promote comprehensive oral health care, especially in multicultural and socio-economically challenged communities.

However, limitations in this study should be acknowledged. The study recruited participants from a pediatric dental practice in an urban community, limiting the generalizability of the findings to non-urban communities or children without a dental home. Additionally, the cross-sectional design of the study precludes establishing causal relationships between social support, knowledge on dental utilization, and dental caries. Future research employing longitudinal or intervention studies would provide a deeper understanding of the causal nature of these associations.

Strengths of this study include a high survey response rate and a large sample size, enhancing the robustness and generalizability of the findings. The practical implications of this study can inform dental practitioners about the significance of social support in discussions about oral hygiene, diet, and treatment plans. Dentists should consider the social support networks of parents and address transportation barriers to facilitate access to dental care for Hispanic children.

## Conclusion

Overall, this study highlights the significance of social support and knowledge on dental utilization in promoting oral health and reducing dental caries rates among Hispanic children. The findings underscore the need for interventions that enhance social support networks, address transportation barriers, and improve functional health literacy among parents. By addressing these factors, oral health disparities in Hispanic communities can be effectively mitigated, leading to improved oral health outcomes for children.

## Data Availability

The raw data supporting the conclusions of this article will be made available by the corresponding authors.
